# The proposal of a modeling methodology for an industrial internet information model

**DOI:** 10.7717/peerj-cs.1150

**Published:** 2022-11-15

**Authors:** Sicong Yu, Ying Huang, Tao Du, Yinglei Teng

**Affiliations:** 1 Technology and Standards Research Institute, China Academy of Information and Communications Technology, Beijing, The People’s Republic of China; 2 Key Laboratory of Internet and Industrial Integration Innovation, MIIT, Beijing, The People’s Republic of China; 3 Beijing Key Laboratory of Work Safety Intelligent Monitoring, Beijing University of Post and Telecommunications, Beijing, The People’s Republic of China

**Keywords:** Information and communication technology, Information model, Modeling method, Industrial Internet, Interoperability

## Abstract

With the large distributed, autonomous, diverse, and dynamic information sources generated in the Industrial Internet area, the information model becomes the critical technology for heterogeneous data interoperability. By establishing unified architecture, mutually agreed communication protocols and standardizing syntax and semantics, the potential of complex data can be released. However, most of the existing information models are isolated in the professional fields, and the interoperability and scope of standards are very limited. In this article, we design a uniform information model for the Industrial Internet, and present a general modeling method which aims to build a standardized organizational framework of information. Specifically, the Industrial Internet information model is first defined, where the seven key elements and value evaluation are devised for information extraction. Then, an optimization approach combining entropy and semantic distance theories is proposed that determines the information organization granularity. Next, as the cross-layer interaction of complex information is very tricky in a tree structure and its modeling cost is extremely high in a mesh topology, the underground root structure is invented for model representation. Finally, the modeling methodology is applied to the ordinary and precision machine tools demonstrating 18.75% and 18.18% modeling cost reduction, respectively, and these two information models are further implemented in a digital machining workshop to verify the effectiveness of the proposed modeling method.

## Introduction

Entering the digital age, the advanced information and communication technologies (ICT) is revolutionizing social life from every aspect. By combining new information and communication technology with manufacturing, global industry has shifted its way to establishing the digital, networked and intelligent industrial production, *i.e.,* Industrial Internet (Li et al., 2020). However, in the industrial domain, the extensive scale connection of dispersed people, machines, objects and other kinds of production factors requires uniform behavior across a variety of data planes, where the data communication and interoperability technologies are to be strongly emphasized.

Due to the heterogeneity of technology and interconnectivity, the true realization of the Industrial Internet ecosystem is currently hampered by multiple dynamic integration challenges (Ali, Ishak & Bhatti, 2021). In the Industrial Internet domain, since interoperability among applications, systems and devices requires agreement on the semantic context and meaning of the data being exchanged (Jaleel et al., 2021), the existing characteristics of diversity and complexity of data make interoperability rather difficult (Reif & Meeus, 2022). Accordingly, a formal information model with scalability by standardizing data across a wide range of industrial field is imperative. Information model technology specifies the static and dynamic behavior of Industrial Internet systems, and it is supposed to provide communication bridges between physical and information spaces (Yu et al., 2020).

Factually, the information modeling is initiated in computer sciences, but emphasized during the process of digital factory. In 1967, the first object-oriented descriptive language *simula67* was put forward, which provided a higher level of abstraction and encapsulation than subroutines, and introduced the concepts of data abstraction and classes information for object modeling (Dahl, Nygaard & Myhrhaug, 1970). Codd E F (Codd, 1970) believed that data does not exist in isolation, and proposed a relationship model to represent data-to-data connections through the collection of tables (Chen, 1976). Subsequently, an object-oriented modeling method (Wong, Mak & Lau, 1999) was proposed. Since its construction extended from simple data to complex information fields, the modeling relationship no longer focuses on single data. After that, extensive research arose from academia to industry focusing on the construction of data relationships. For example, the entity-relationship(E-R) model was proposed to provide methods for complex data relationship construction (Cohen & Gil, 2021; Wang et al., 2019; Zhang, Ma & Cheng, 2016). However, complex and diverse features make information extraction very hard. For this reason, a bottom-up modeling method from one-metadata to binary derivation was designed (Li & Qiao, 2007), which began with the collection and sorting of essential data, gradually defined accurate information elements and established their association, after that an information model could be built. In these methods, the basic data defined related with a specific chosen domain has respective application constraints of the maximum range, which means that there is a large workload for the modeling of complex information.

With the information interoperability going complex, the semantic relationship modeling method with the ability to construct the association relationships among complex attributes of entity objects is highly desired (Yang et al., 2022; Bertoli et al., 2022). Furthermore, ontology and entropy theories were introduced to optimize the semantic model structure (Dai et al., 2021; Yu, Thom & Tam, 2009). The ontology was initially designed for describing knowledge relations and widely used in the area of the database. Based on the ontology theory, it was believed that there might be some similarity between two terms facing the same object, and the aggregation of two terms could be achieved according to semantic similarity (Gruber, 1993). In particular, to overcome problems due to semantic heterogeneity and to support interoperability with external systems, ontologies could be used as a conceptual schema to represent both data sources to be integrated and the global view to be built. According to that, an approach was presented for the semantic integration of heterogeneous data sources (Fusco & Aversano, 2020). Meanwhile, information entropy has been investigated for a long time in the information communication field (Kuang, 2021; Li et al., 2018). One of its applications is to measure the information granularity (Qian et al., 2011), which can also be used in the semantic information organization.

As a series of information model standards were developed, the method of information modeling gradually became mature (Genesereth & Ketchpel, 1994; Barreiro et al., 2003; Paukera et al., 2016; Li & Tang, 2021). Based on the ISA95 standard, a layer-by-layer decomposition modeling method was designed (ANSI/ISA95), which could be used to define the descriptive terms in the integration of business systems at enterprise level. UML, XML, JSON and other data format description languages have been successively used as international standards, which provided the basis for the interconnection and interoperability of data (Abdalazeim & Meziane, 2021; Larman, 1998; Ma et al., 2003; Kristensen, 1999; Singh & Sachdeva, 2020; Bourhis, Reutter & Vrgoč, 2020). In 1996, the OPC specification version 1.0 was released (Markus & Branko, 2009). In 2000, the Ecl@ss Association was established, and the systematic research work on semantic standards was officially launched, supporting the complex interconnection network of the standardized industry description of the information (Hepp, Leukel & Schmitz, 2007). Pack ML, Automation ML, Instrument ML and other standard projects have promoted the information model application in packaging, engineering automation, and instrument domain (Barbieri, Battilani & Fantuzzi, 2015; Ye et al., 2018). By utilizing the OSI 7 layer model (Open Systems Interconnection model), an open application programming interface (API) for injection molding machines (IMMs) was developed, which had the potential to be applied with different IMMs to log and set the necessary process parameter values (Ogorodnyk et al., 2020).

In general, the researches on information model technologies are mainly focused on the area of information management, database designing, engineering automation etc. Furthermore, there is no mutual recognition among standards, which brings challenges to the applications of these information models in the Industrial Internet. Existing standards of modeling methods mainly concentrate on a particular field or industry, resulting in firmly professional but isolate status. To accelerate the interoperability in standards, the Industrial Internet Information Model (3IM) is firstly proposed in this article, and a general information modeling method is studied for the Industrial Internet. Specifically, an optimization method for information organization is invented based on the ontology and entropy theories. Moreover, based on the modeling method, two 3IMs are developed for ordinary and precision machine tools, respectively. Finally, the research results of this article are verified in an application scene of a digital machining workshop.

## Description of internet information model

### Definition of 3IM

In this article, 3IM is defined as a kind of generalized semantic expression in the information space of the Industrial Internet. By specifying the description of static and dynamic behavior in Industrial Internet systems, 3IM can be used to realize the seamless communication among various physical objects and heterogeneous systems. In the Industrial Internet area, all the information from the elements, value and supply chains can be modeled, which provides a standardized way of capturing the conceptual manufacturing process and enabling interoperability. For an industrial system, the elements refer to devices, applications, systems, products and so on. A value chain is to describe the information assets during the whole manufacturing process involving a product from conception to distribution, *e.g.*, procuring raw materials, manufacturing functions, and marketing activities. A supply chain is a network of producing and delivering a product or service, *i.e.,* from the very beginning stage of sourcing the raw materials to the final delivery of the product or service to end-users.

**Figure 1:**
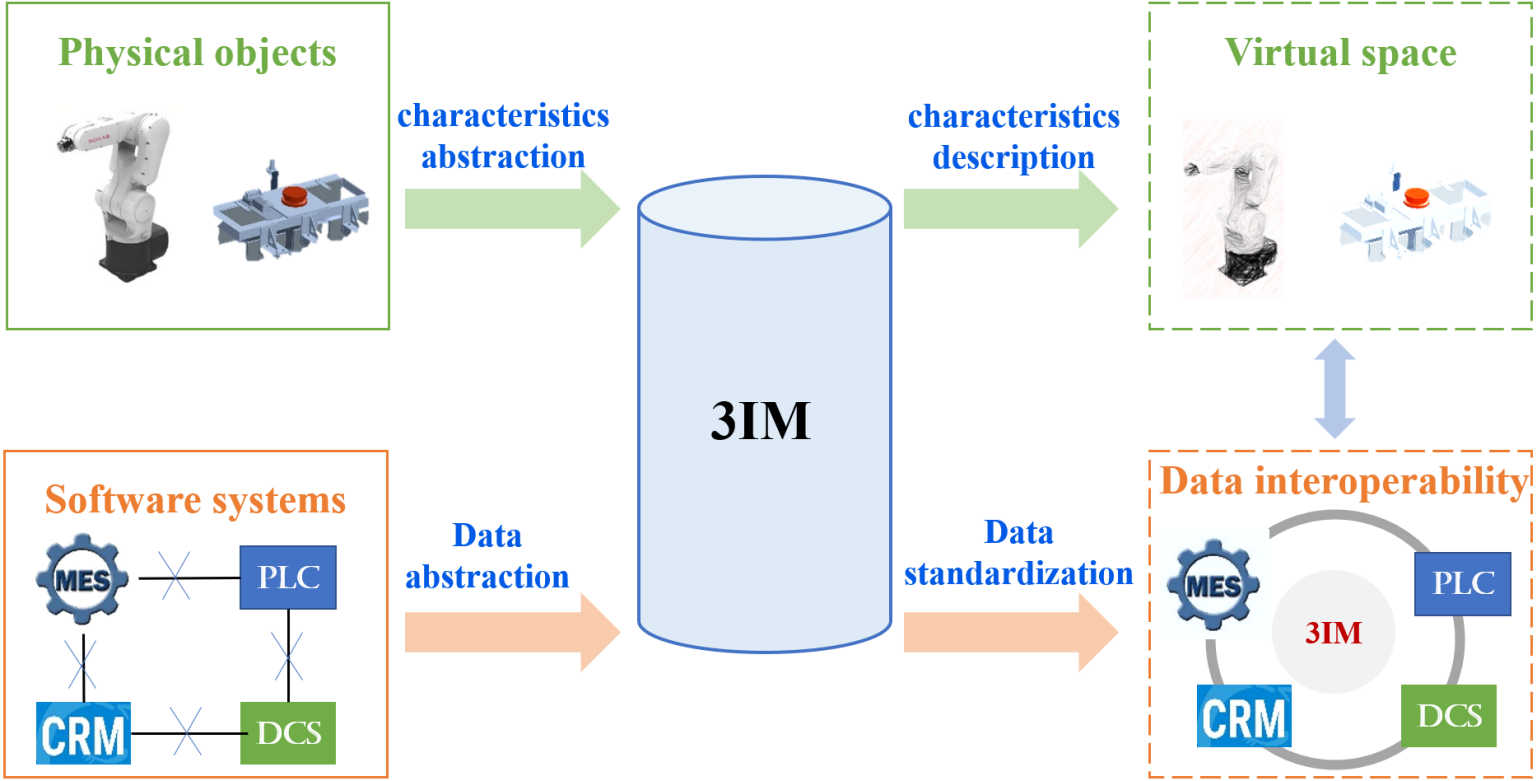
The function diagram of 3IM.

3IM presents a top-level model architecture through summarizing and abstracting the modeling elements considered by the existing information models, *e.g.*, OPC UA information model, Automation ML, which is much more general because it does not involve configuration of professional attributes. As the 3IM is developed on the basis of the existing information models, it is compatible with them. On the other hand, when it is applied in the specific domain, 3IM can be further professionalized and developed by defining corresponding modeling elements. In virtue of its versatility and scalability, 3IM can be applied widely in the Industrial Internet for heterogeneous data interoperability. As shown in Fig. 1, both the information from physical objects and software systems can be modeled in 3IM. By describing the shape, color, type, manufacturer, status and other characteristics, a physical object can be completely represented through 3IM in virtual space. Since there are different types of manufacturing software developed by different suppliers, they may contain incompatible data formats that obey different standards. To realize the collaborative work across systems and maximize the value of industrial data as well, it is necessary to standardize the industry data model syntactically and semantically in 3IM. Accordingly, not only the data interoperability among different enterprise applications can be ensured, but also the digital twins at scale can be achieved by integrating and synchronizing the physical and virtual counterparts tightly.

### Reference architecture of 3IM

**Figure 2:**
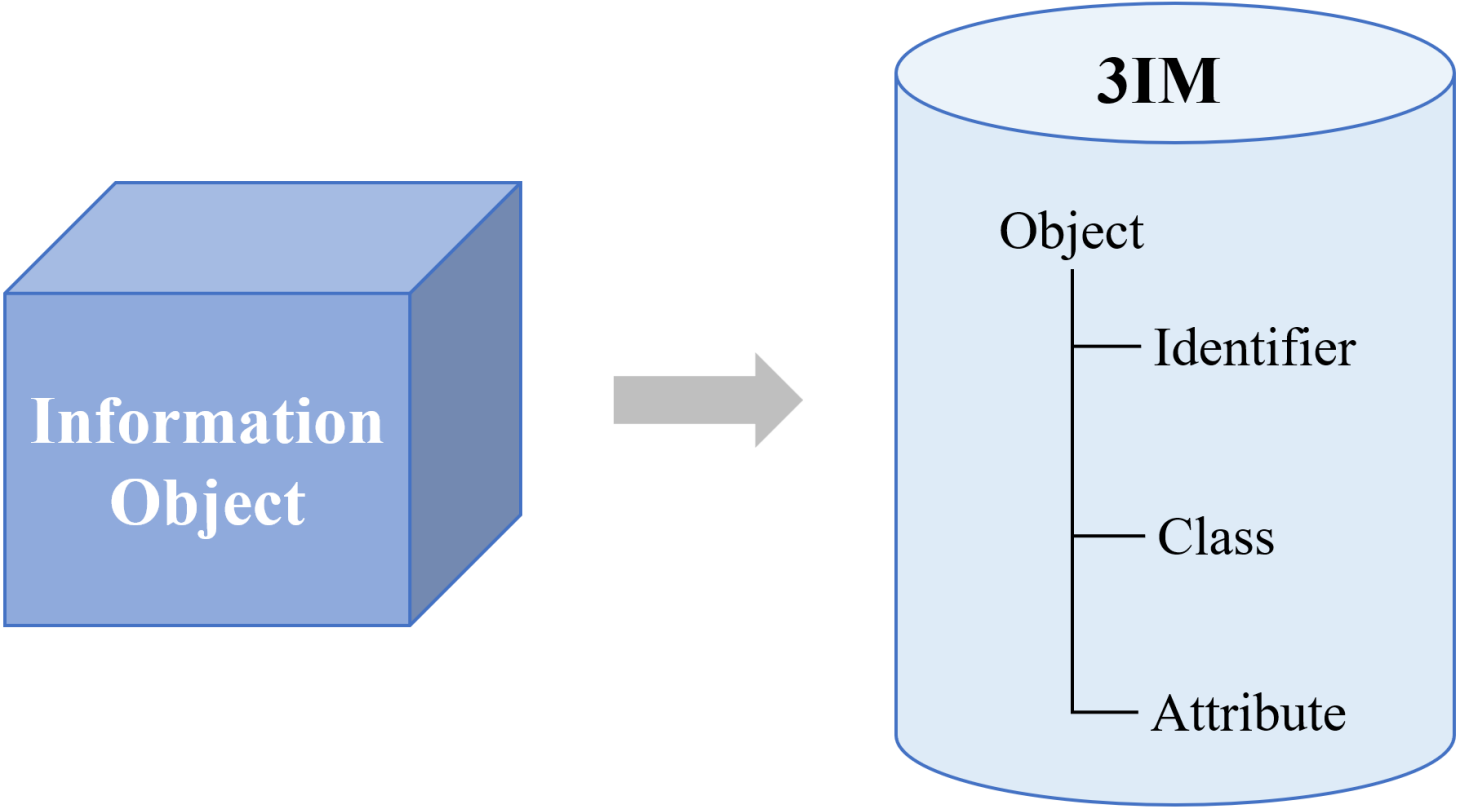
Reference architecture of the 3IM.

To ensure the massive information effectively organized, 3IM adopts three basic elements, including *identifier*, *class* and *attribute* in the modeling framework, as shown in Fig. 2. Typically, the identifier is defined with the model, data, and properties. To remove the ambiguity, each identifier is assigned globally unique and persistent. For ease of reference, each model should have at least one identifier. The class is a set, collection, group, or configuration containing members regarded as having certain traits in common. The attribute is a professional description for an object, which includes configuration attribute, process attribute, and static attribute, such as status, shape, service, action, location and so on.

**Figure 3:**
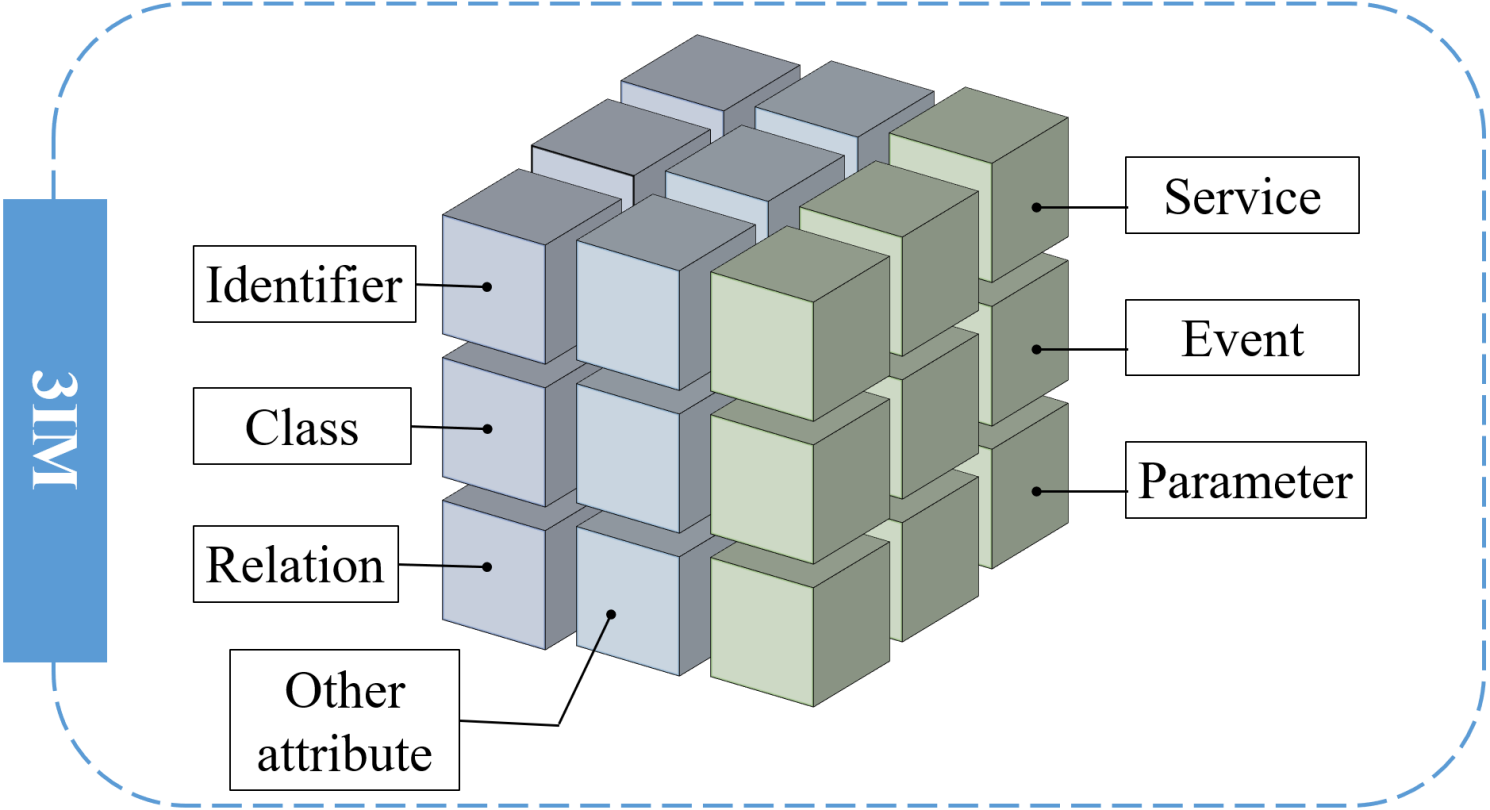
Seven key elements for information modeling.

To facilitate the modeling features extraction, the attributes of *relation*, *event*, *service* and *parameter* are defined exclusively, as shown in Fig. 3. Service is used to describe the actions of an object, such as to access and control data. Event is to specify the various types of event-trigger functions generated by the running-on services, such as launching or receiving instructions, while parameter is the numerical or other measurable factor that defines the system value, *e.g.*, input and output values, object size parameters, etc. Combined with the above three essential factors, we construct the seven key elements which are vital for the information extraction of the industrial information model.

### Application rules of 3IM

The 3IM plays different kinds of roles when applying to different industrial internet layers. At the device level, an information model serves as an attachment that integrates data generally for every entity, which makes the information available to different areas of the business with respect to their specific applications. When 3IM works in a system, it goes beyond the boundaries of an organization, and mainly focuses on building a communication bridge among various components. After that, the ability of two or more business units to exchange information and the mutual usage of distributed information will be realized, which supports the integration of the manufacturing system by standardization at scale.

**Figure 4:**
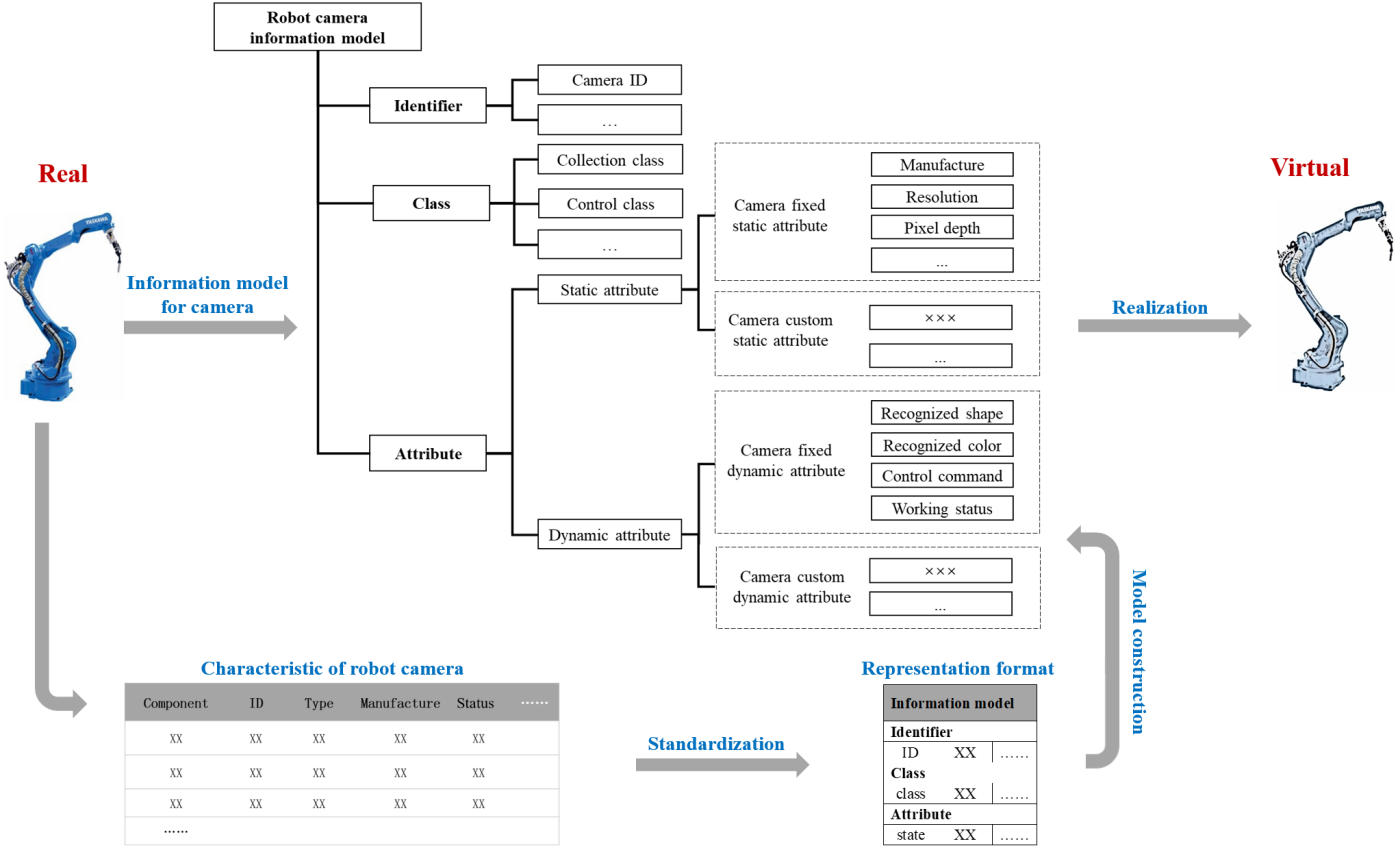
An example of a robot camera information model.

Figure 4 presents an example of a robot camera information model in a manufacturing system. Since the upstream and downstream objects of a robot camera may come from several suppliers, it needs a digital description in a semantic way. In particular, the information model can be generated as an organized structure of information requirements according to the domain context. For a single entity, information is extracted, and a simple information model is established according to the needs of information interaction. As for a complex entity, multiple simple information models can be composited to satisfy the multidimensional information interaction.

### Modeling method and optimization theory

To provide a general modeling methodology, the modeling process of 3IM can be divided into three steps, *i.e.,* information extraction, information organization, and model representation. Since the source information generated from diverse industrial systems is various and even complex, it needs to be extracted first in relevance to a specific topic. Next, we propose an information organization method regulating the dispersed distributed source elements into a tight economical structural space. Finally, a novel model representation framework is established that combines the advantages of the tree and network structure.

### Information extraction method

Information extraction is the process that generates the retrievable and editable information related to a specific requirement from unstructured textual sources. Regarding the various distinct features of source information elements, we apply the enumeration method for information extraction. Firstly, the functional composition of an object in each application dimension should be clarified. For example, if the purpose of modeling is to manage the equipment operation, the information extraction should focus on the equipment operation status, while if the modeling purpose is to assemble manufacturing apparatus, the information of equipment function and appearance should be included in equipment specification. After that, the whole object is decomposed from top to bottom to obtain several relatively independent analyses. Regarding the problems that the model needs to serve, the seven key elements of 3IM can be used to enumerate various features of each object, including identifier, attribute, event, service, parameter, and relation. It is worth noting that the modeling elements in the industrial domain, such as part name and part number, need to be reasonably defined and named for clarification.

**Figure 5:**
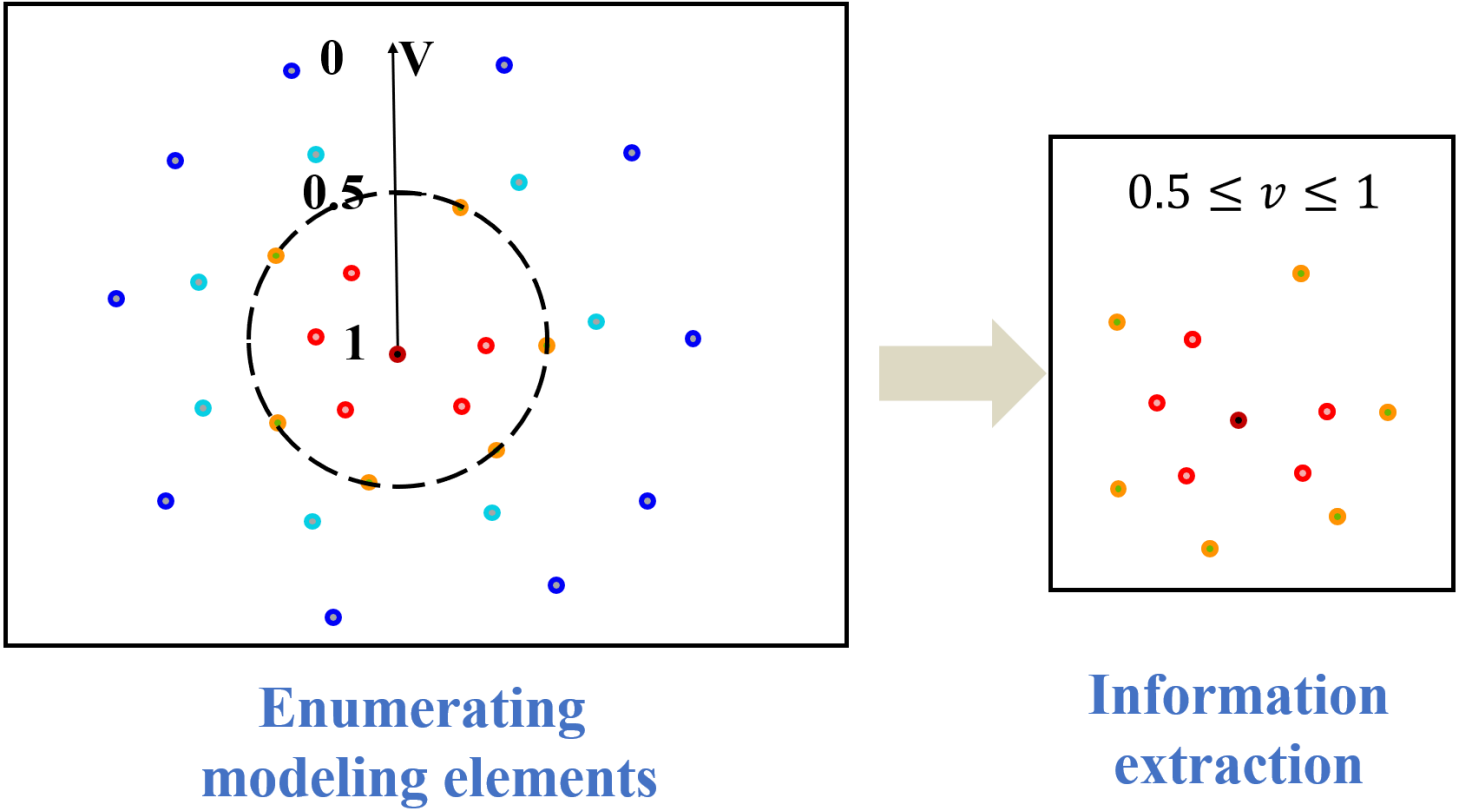
Value evaluation methodology.

Secondly, to classify the composition information of an entity, even if its subordinate branch objects only constitute the zero-test part of the superior object, the level-by-level division process can be extended to infinity because the composition information of the entity is endless (Chirok & Dukpa, 2020). The information modeling requires appropriate modeling elements selection criteria and value evaluation methods, which are related to the foundation of 3IM modeling. Therefore, a modeling value evaluation mechanism is introduced in information extraction. With the help of modeling value evaluation, the composition information of an entity can be regarded as an extensive but limited system. According to its importance to the model, the modeling elements can be divided into four categories for the multi-dimensional multi-domain modeling. Specifically, the value (*v*) of the modeling element is predefined empirically based on four categories given in reference (Li & Liu, 2001), (1) for the modeling elements definitely to be used, *v* = 1, where *v* is the modeling value indicating the significance of the modeling elements in the modeling process; (2) for the modeling elements most likely to be used, 0.5 < *v* < 1; (3) for the modeling elements not to be used now, but may be used in the future, which means considering value is less than not considering value, 0 < *v* < 0.5; (4) for the modeling elements definitely not used, *v* = 0. Here, 0.5 is a predefined threshold which can be devised according to the statistical calculation of the actual industrial conditions. As shown in Fig. 5, only the modeling elements whose modeling value are greater than or equal to 0.5 are selected in this article.

### Optimization method for information organization

**Figure 6:**
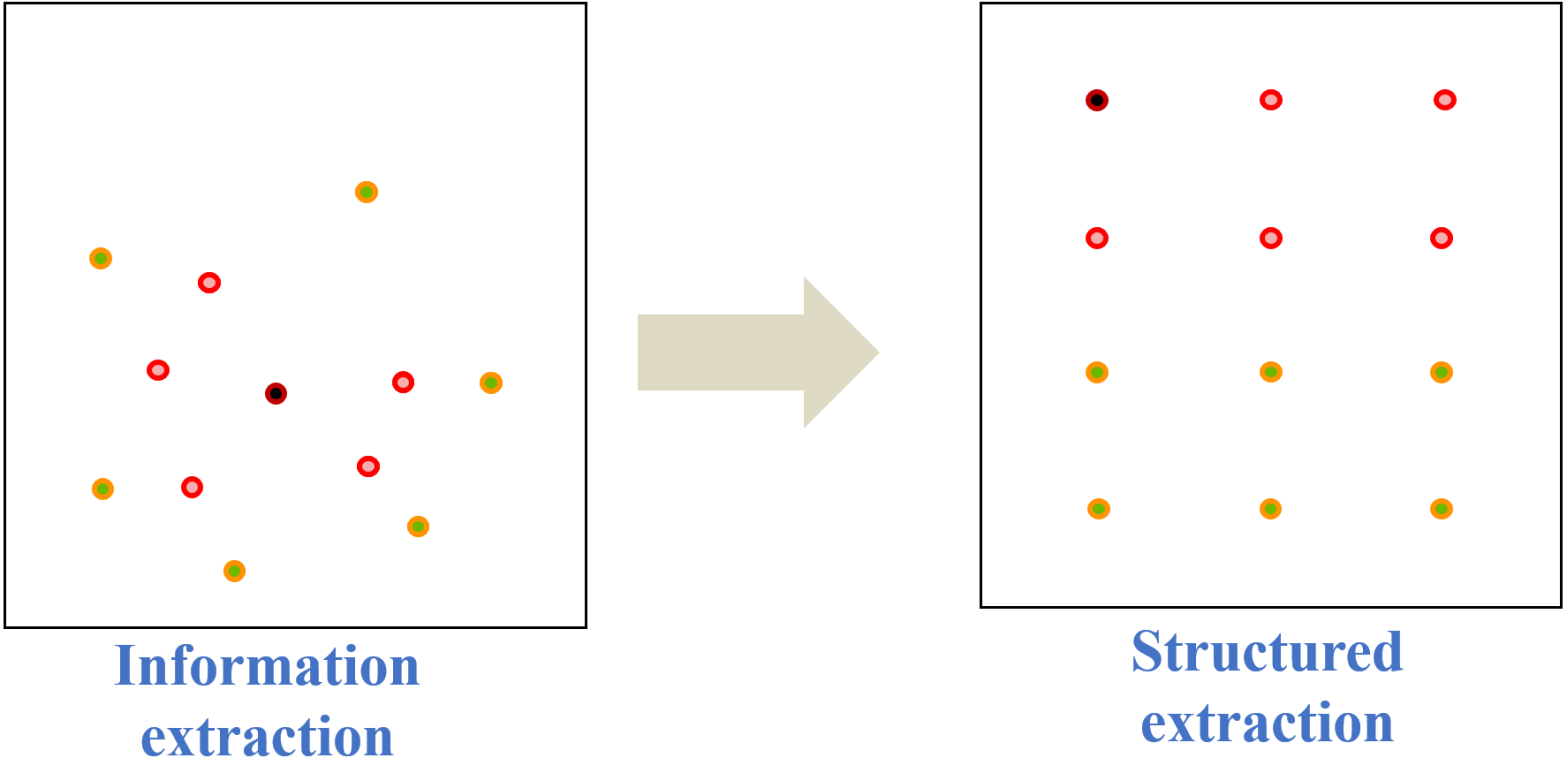
A diagram of structure information.

After the modeling elements are extracted, as shown in Fig. 6, they can be structurally organized according to special requests based on the existing conditions. By adding deterministic and indeterminate industrial relationships for the modeling elements according to the common sense, the modeling elements are arranged into a cluster of related tasks that can be handled by certain individuals or groups. As the modeling elements are structured, we can calculate the semantic similarity of two adjacent elements from their inherent relationship structure. Then, the elements whose semantic similarity reaches a certain threshold could be aggregated to reduce the number of modeling elements, that is, the structure of 3IM models can be further optimized.

Although information extraction specifies finite structured factors, these collections may still be too complex to assemble an appropriate information model, especially regarding the storage and search schema. On the contrary, if the reserved information lists are too concise, it may lead to an insufficient expression for the model representation. Therefore, the optimization method is crucial to build the information model which needs to achieve a balance between the model complexity and information completeness. With these considerations, the optimization process of the 3IM is elaborated as follows.

**Figure 7:**
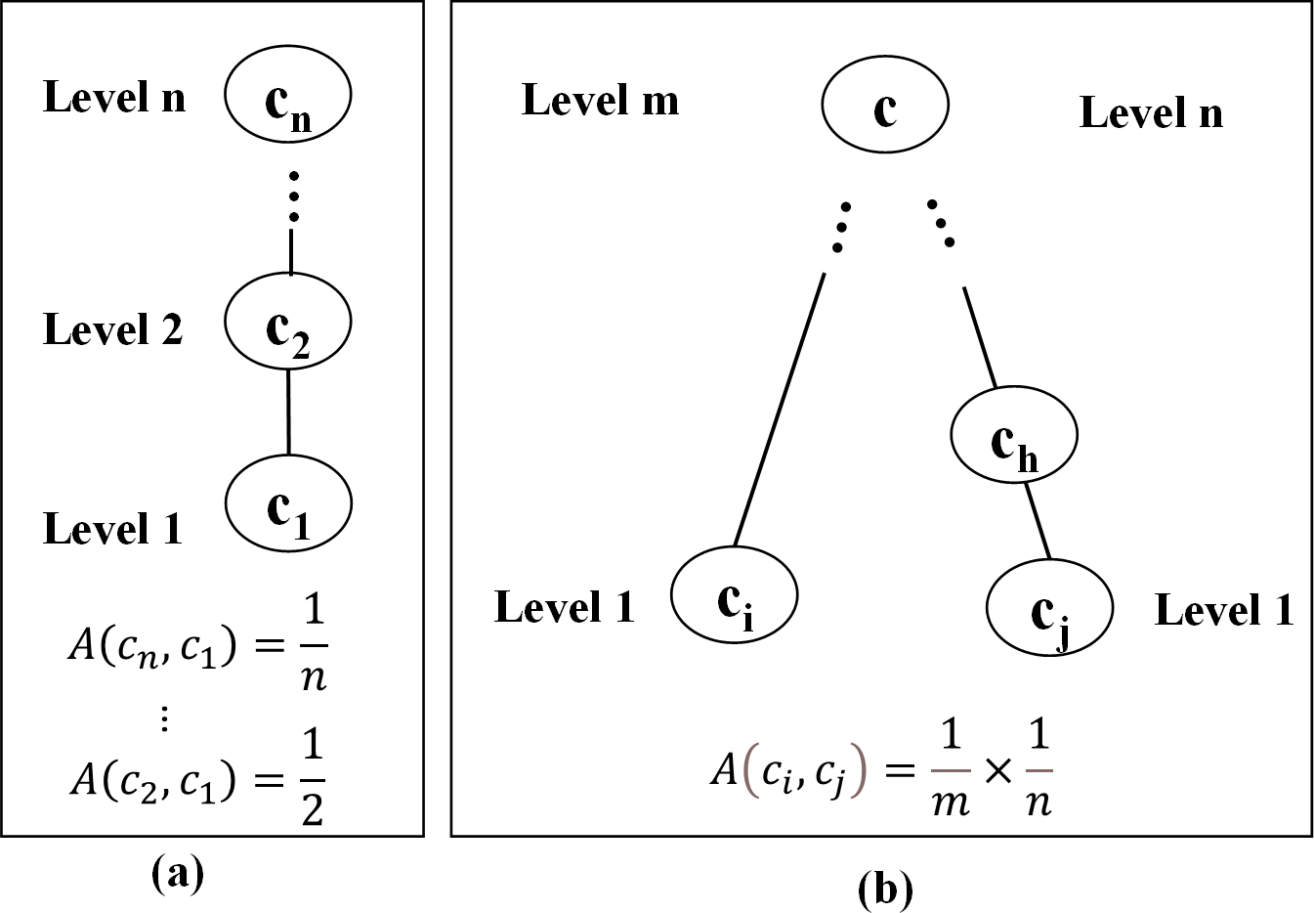
Calculation method of relevance value.

Firstly, the modeling elements should be classified based on the vertical and hierarchical relationships among attributes. For the convenience of classification, the semantic similarity value is defined based on the on-line lexical database building method of WordNet (Miller et al., 1993), which is developed from the ontology theory. Assume the semantic similarity value between elements ranges from 0 to 1. Figure 7 illustrates the way how to calculate the semantic similarity. As there is a connection path between two modeling elements in a conceptual system, it means they are related. For example, since *c*_*n*_ is the mother semantic node of *c*_1_ upper *n* levels, the semantic similarity between these two nodes is set as $A \left( {c}_{n},{c}_{1} \right) = \frac{1}{n} $. Similarly, when the upper *m* layers of *c*_*i*_ and the upper *n* layers of *c*_*j*_ have the same mother semantic node, set $A \left( {c}_{i},{c}_{j} \right) = \frac{1}{m} \times \frac{1}{n} $. Especially, if there is no connection path between two modeling elements, *n* can be considered as ∞, and their semantic similarity value becomes 0 (*A* = 0). Conversely, when the semantic similarity between two concepts is 0, *n* is regarded as 1, *i.e.,* the semantic similarity value is 1 (*A* = 1). Accordingly, the semantic topology in the form of hierarchical clusters is established indicating the affinity among the modeling elements.

Next, proper modeling elements need to be selected to assemble an effective information model. For a 3IM, the information entropy can be used to determine the dispersion of information classification (Teng et al., 2012). From the perspective of entropy theory, a large information entropy means a tiny information granularity, that is, more modeling elements, and detailed characteristics are required in order to describe the physical entity. However, too tiny granularity makes a rather bulky model, which will result in a too complex model and do harm to data response speed. Therefore, an appropriate semantic similarity threshold should be chosen according to a suitable information classification principle. Based on the concept of semantic similarity and information entropy, the modeling elements can be aggregated, and the optimal particle information in the model can be generated combining the functional requirements and the 3IM complexity. For example, in the later examples of 3IM, the default semantic similarity value is set to 0.25 on the basis of empirical observations, *i.e.,* the modeling elements could be clustered when their semantic similarity value is greater than or equal to 0.25. Note that this default value can be adjusted dynamically with respect to the characteristics of modeling objects and the specific interoperability application.

### Model representation framework

**Figure 8:**
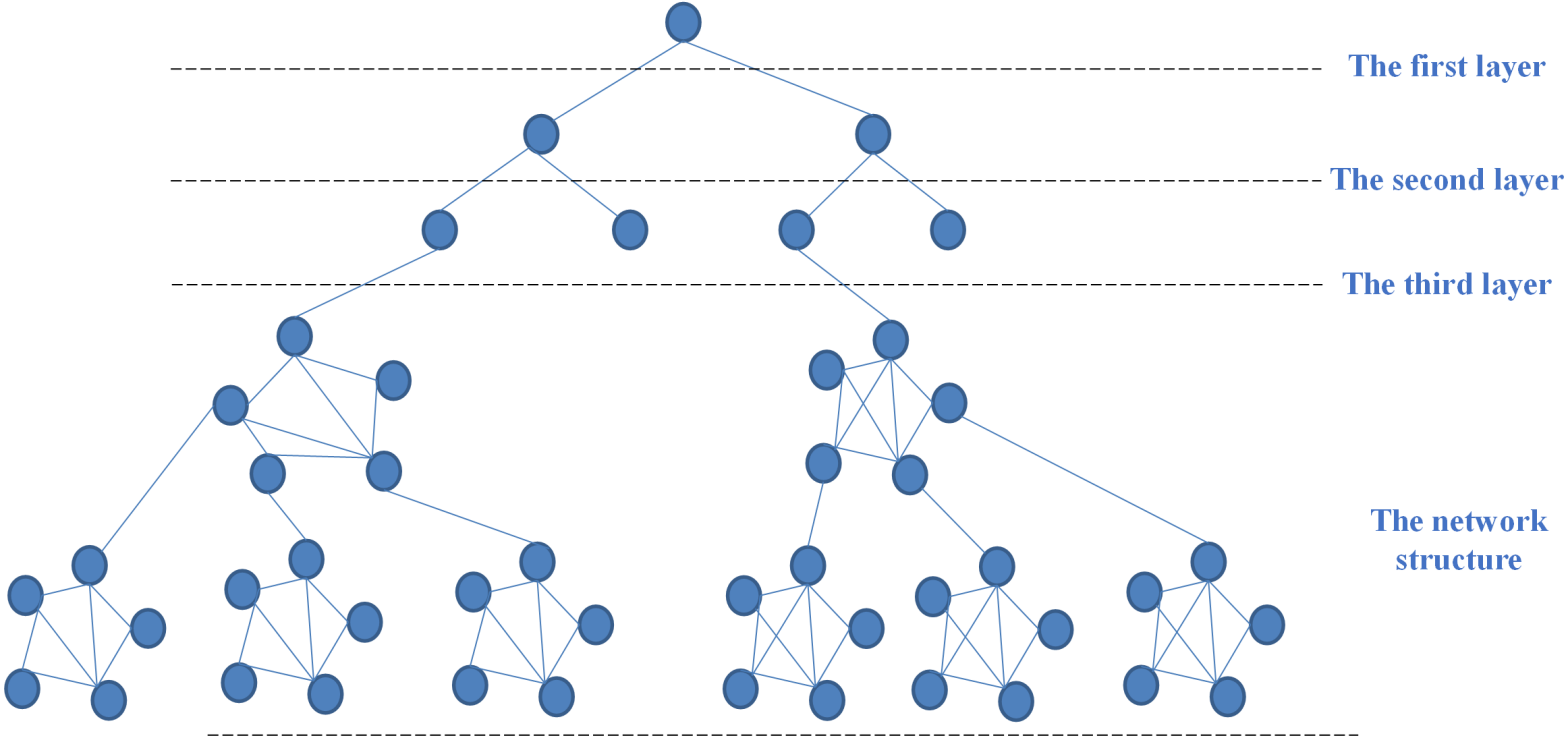
The underground root structure.

For maximizing the value of the organized information, the 3IM should be represented with a suitable structure. Considering topology complexity, this study proposes a model representation framework, called underground root structure (URS) by combining both the tree structure and network structure. Unlike the hierarchical characteristics and strict classification in the tree structure, the last layer of URS develops a mass of roots to express the complex, unstructured and even interlocking relationship of extended attributes in the information model. The framework of URS is shown in Fig. 8. Specifically, the first three layers adopt the tree structure. Starting from the fourth layer, since the information with associated relationships appears as a laterally branching network, it constructs a systematic flat form that avoids the over-hierarchical tree. Accordingly, the underground root structure allows connections and semantic relations realized horizontally or cross-layered as needed.

**Figure 9:**
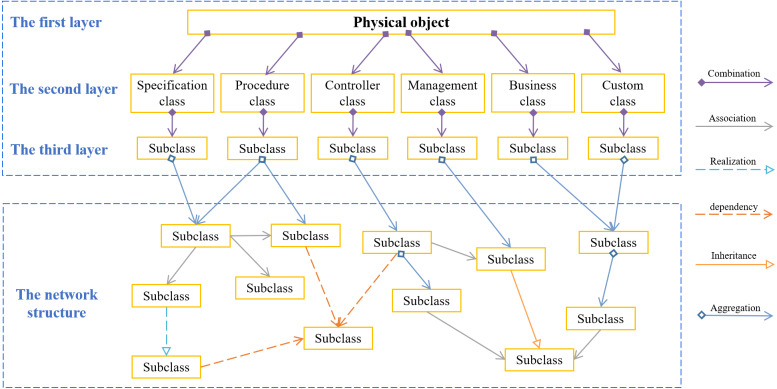
An example based on underground root structure.

A 3IM for a physical object based on URS is illustrated as in Fig. 9. This structure defines a basic information model framework. The first layer is an object class, and the second layer includes a specification class, process class, controller class, management class, business class, and custom class. Here, custom class refers to some attributes that are not easy to be abstracted due to expert knowledge. The subclasses in the third layer are constructed by abstracting the modeling elements in the fourth layer. For some scenarios, the modeling structure may only have the first three layers that the relationship of the modeling elements in these layers is combination. For others, the modeling structure contains at least three layers, thus the modeling elements above the 3rd layer should be the remainder five relationship. Moreover, multiple URS can be combined for more complicated information modeling.

Besides, the behavioral relationships indicating how each class is linked with others during model execution should also be reflected in 3IM, including aggregation, combination, dependency, association, inheritance, and realization. The *aggregation* simply means that one modeling element is linked in some way to another one, which can be represented by an arrow showing the flow of control. The *combination* reflects the relationship between the whole and the part, which is a special case of association relationship, in this relationship the whole and the part is indivisible. For two relatively independent modeling elements, where one element is responsible for constructing an instance of another one, or depends on the service of another one, the *dependency* relationship between them is mainly reflected. The dependency relationship is passed into the dependent class in the form of parameter variables. The *inheritance* refers to the ability of a class to inherit the functions from another class, while the *realization* means a relationship between two elements that one element specifies behavior and the other implements or executes.

### Example verification

Ordinary machine tool (OMT) and precision machine tool (PMT) are common devices in the Industrial Internet domain. Compared with the OMT, the PMT is the important device for high precision mass machining, which involves more information elements. In this study, two kinds of 3IMs for the OMT and the PMT are built to verify the effectiveness of the modeling method. Firstly, based on the seven key elements, various controller information of the machine tools is extracted. From the basic description, *e.g.*, the information of manufacturer, factory batch, serial number, equipment type, production date, running status, running time, alarm status, etc., to functional definition *e.g.*, Computer Numerical Control (CNC) system, CNC controller, auxiliary system, PLC controller, cooling system, sensor, etc., all these factors can be considered as the modeling elements.

There are many known industrial relationships based on common sense, such as the production logic, business organization, management rules, customer service. In this study, these modeling elements could be associated according to the business organization viewpoint. For a machine tool, the basic inherent information usually contains manufacturer, factory batch, serial number, equipment type and production date. As the machine tool is an operating equipment, the running status and working status can be used to describe its dynamic information, which includes running time, alarm status, and material in and out from warehouse. Meanwhile, the machine tool can also consist of CNC system, CNC controller, auxiliary system, PLC controller, cooling system, lubrication system, electric servo system, sensor, spindle drive, spindle motor, hydraulic system. Initially, regarding different concepts, the semantic similarity among terms is 0, they are certainly not the same class. From this perspective, the whole modeling elements are categorized as two sets of MTA (attributes of the machine tool) and MTC (components of the machine tool), which represents the description attributes and the components of the machine tool, respectively. Furthermore, the MTA contains static and dynamic description information, the MTC has key and auxiliary components. Specifically,

#### The ordinary machining machine tool

MTA = {manufacturer, factory batch, serial number, equipment type, production date, running status, running time, alarm status, working status},

MTC = {CNC system, auxiliary system, hydraulic system, lubrication system, spindle drive, spindle motor}.

#### The precision machining machine tool

MTA = {manufacturer, factory batch, serial number, equipment type, production date, running status, running time, alarm status, working status, material in and out from warehouse},

MTC = {CNC system, CNC controller, sensor, spindle drive, spindle motor, PLC controller, auxiliary system, cooling system, lubrication system, electric servo system, hydraulic system}.

**Figure 10:**
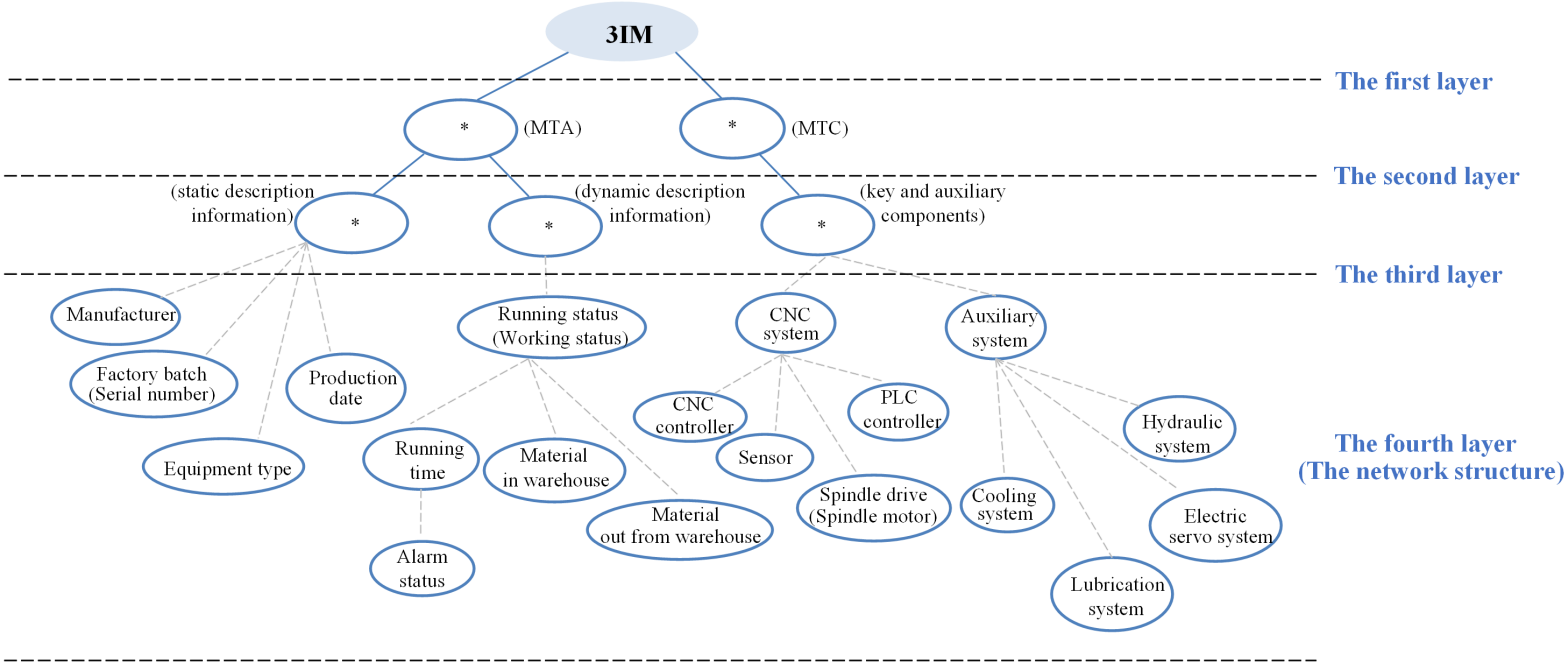
The initial architecture of 3IM for the two machine tools.

After that, the initial URS of 3IM can be drawn as Fig. 10, where the * is used to represent subclass in the first three layers while there is currently no suitable word to describe them. As the modeling architecture is built, the * will be finally replaced by certain subclass, such as specification class, procedure class, controller class, and so on. Since the initial form in the network layer whose relationships are not real, the modeling elements are connected with dash lines.

There are different application scenarios for the two machine tools, thus the considered modeling elements should vary from case to case. In this case, the modeling value of modeling elements greater than or equal to 0.5 should be retained.

For the OMTs with low machining requirements, the factory batch, serial number, and equipment type are the must-be-considered components, which means that their modeling values are 1. The manufacturer and production date may be used in machine operation and maintenance, therefore, 1 > v_manufacturer_ > 0.5, 1 > v_production date_ > 0.5. As for the running status, working status, running time, and alarm status, they are the necessary descriptions of the working process that must be used, that is, their modeling values are 1. The information of material in-and-out from warehouse cannot be used now, 0.5 > v_material warehousing_, v_material out of the warehouse_ > 0. The numerical control system is improved by a simple drive motor, and the hydraulic and lubrication systems are used for auxiliary functions, thus the modeling values of the controller and electric servo system are both 0, which means they are not suggested as necessary for OMT modeling. The sensor, PLC controller and cooling system cannot be used now, but may be used in the future, so 0.5 > v_sensor_ > 0, 0.5 > v_PLC_ > 0, 0.5 > v_cooling system_ > 0.

For the PMTs with high machining requirements, the factory batch, serial number, and equipment type must be considered, then their modeling values are set 1. The manufacturer and production date may be used in machine operation and maintenance, even though they are not necessarily included, accordingly, 1 > v_manufacturer_ > 0.5, 1 > v_production date_ > 0.5. Since the information of running status, working status, running time, and alarm status are the essential descriptions of the working process, that is, they must be considered and their modeling values are 1. The information of material in-and-out from warehouse is definitely needed for the machine tool to schedule materials, then v_material warehousing_ = 1, v_material out of the warehouse_ = 1. To ensure the control accuracy, the numerical control system is designed separately, including the controller, spindle drive, spindle motor, sensor, and PLC controller. The electric servo, cooling and lubrication system should be used for auxiliary function, v_CNC_ = v_spindle_ = v_PLC_ = v_sensor_ = v_electric servo_ = v_cooling system_ = v_lubrication system_ = 1. Correspondingly, the modeling value of the hydraulic system is 0.

**Figure 11:**
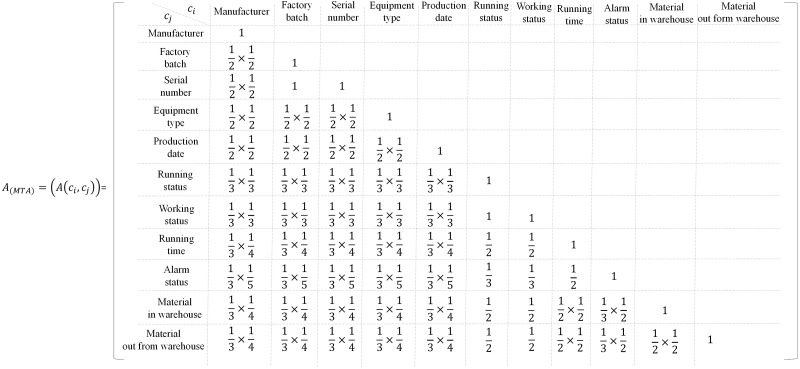
The relevance value between every two modeling elements in MTA.

Next, the semantic similarity value between every two modeling elements in MTA (*A*_*MTA*_) is calculated as Fig. 11. In this case, the workload of the selected modeling elements to be analyzed is acceptable for both manual and automatic, and considering the validity of the research results, the modeling elements with semantic similarity values greater than or equal to 0.25 (*A* ≥ 0.25) are clustered. The information of equipment type, factory batch, serial number, manufacturer, and running status can be used to describe the machine tool. They are static attributes and dynamic attributes of machine tools, respectively. Their upper one layer has no common semantic node, nevertheless, they have the same semantic node in the machine tool layer. For the information of manufacturer and running status, assuming that their upper two layers have the same mother semantic node, then maxA(c_*i*_,c_*j*_) = A(manufacturer, running status) = 1/3 × 1/3 = 1/9 < 0.25.

For the working status and running status, A(working status, running status) = 1 > 0.25. Therefore, the working status and the running status are clustered as the running status, and the working status is deleted. Since A(factory batch, serial number) = 1 > 0.25, the serial number is retained, and the factory batch is deleted. A{A2 4(manufacturer, serial number, production date, equipment type)}= 1/2 × 1/2 = 0.25, therefore, the manufacturer, serial number, production date and equipment type can be clustered into one category. A(running status, running time) = 0.5 > 0.25, A(running status, material in warehouse) = 0.5 > 0.25, A(running status, material out of warehouse) = 0.5 > 0.25, therefore the running status, running time, material in and out from warehouse can be clustered into running status. A(running time, alarm status) = 0.5, the running time and alarm status are clustered as running time. After that, the following modeling elements (MTA^(ordinary)^) are retained for the OMTs, MTA^(ordinary)^ = {manufacturer, serial number, equipment type, production date, running status, running time, alarm status}= {manufacturer, serial number, equipment type, production date} + {running status}= {manufacturer, serial number, equipment type, production date} + {running time}. The following modeling elements (MTA^(precision)^) are retained for the precision machining machine tool, MTA^(precision)^ = {manufacturer, serial number, equipment type, production date, running time, alarm status, running status, material in warehouse, material out of warehouse}= {manufacturer, serial number, equipment type, production date} + {running status}= {manufacturer, serial number, equipment type, production date} + {running time, material in warehouse, material out of warehouse}. The serial number is unique, and it can be named as the identifier. As there is no horizontal relationship between manufacturer, equipment type, production date, and identifier, and there is no mother semantic node upper them, they can be abstracted as subclassed in the third layer. According to the inherent attributes of the machine tool, they can be clustered and named as specification class. Similarly, running status can be abstracted as a subclass of the third layer and then clustered into process class.

**Figure 12:**
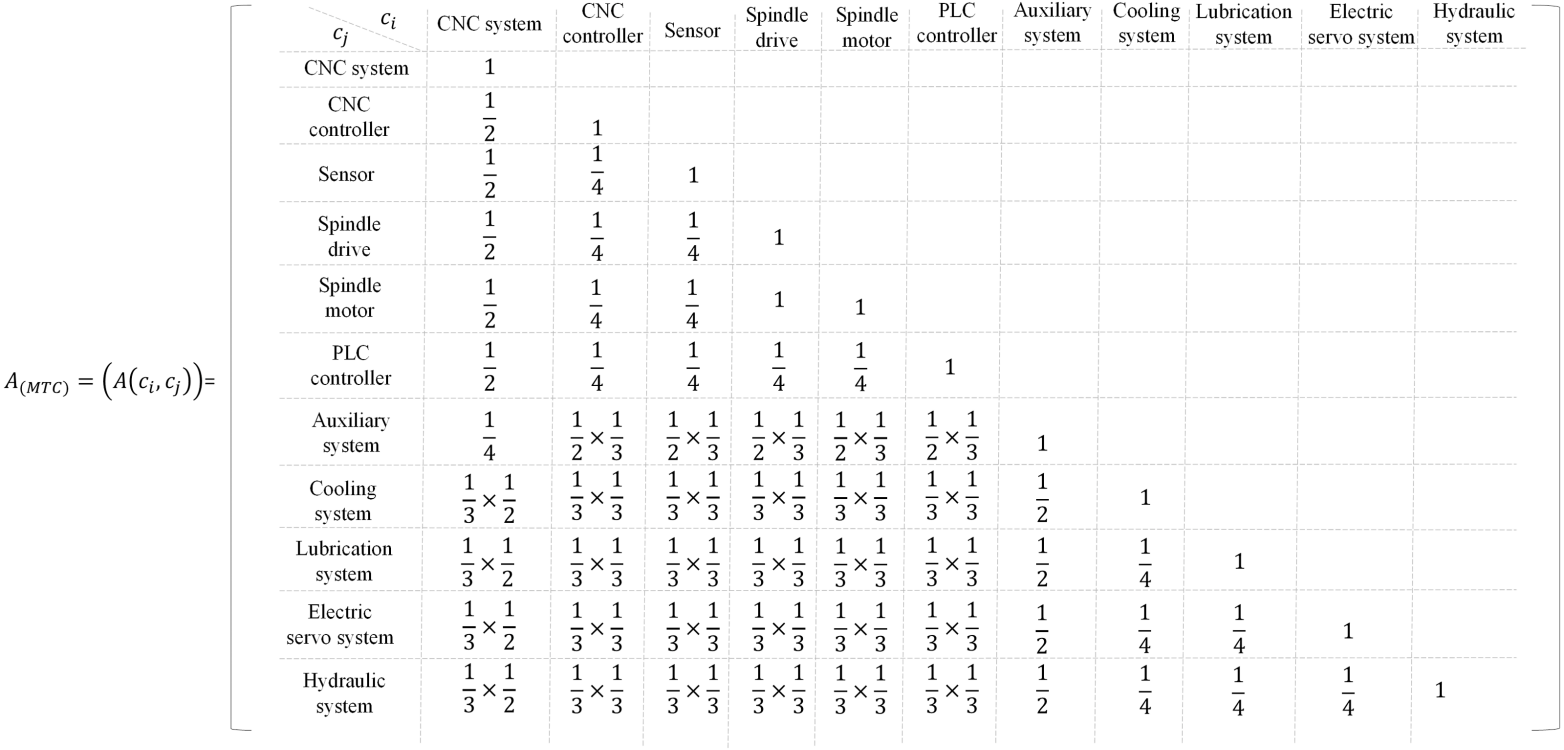
The relevance value between every two modeling elements in MTC.

The specific calculation matrix of MTC (*A*_MTC_) is as Fig. 12, for the ordinary machining machine tool, A(CNC system, auxiliary system) = 1/2 × 1/2 = 0.25, the CNC system and auxiliary system can be clustered into control class. A(spindle drive, spindle motor) = 1, the spindle drive and spindle motor are clustered into spindle motor. A(CNC system, spindle motor) = 0.5, A(CNC system, sensor) = 0.5, the CNC system, spindle motor and sensor are clustered into CNC system. Moreover, the CNC system includes the spindle motor and sensor, and the relationship cannot be broken. A(auxiliary system, hydraulic system) = 0.5, A(auxiliary system, lubrication system) = 0.5, A(auxiliary system, cooling system) = 0, A(auxiliary system, electric servo system) = 0. Therefore, the auxiliary system, hydraulic system and lubrication system are clustered into auxiliary system. The retained modeling elements can be further optimized (MTC^(ordinary)^), MTC^(ordinary)^ = {CNC system, CNC controller, auxiliary system, PLC controller, cooling system, hydraulic system, lubrication system, pneumatic system, sensor, spindle drive, spindle motor}= {CNC system} + {auxiliary system}= {sensor, spindle motor}+ {lubrication system, hydraulic system}. For the precision machine tool, A(CNC system, auxiliary system) = 1/2 × 1/2 = 0.25. The CNC system includes motor, controller and other spare parts, A(spindle drive, spindle motor) = 1, spindle drive and spindle motor are clustered into spindle motor. A(CNC system, CNC controller) = 0.5, A(CNC system, sensor) = 0.5, A(CNC system, spindle motor) = 0.5, A(CNC system, PLC controller) = 0.5, CNC system, CNC controller, sensor, spindle motor, and PLC controller are clustered into CNC system. The CNC system includes the CNC controller, sensor, spindle motor and PLC controller, and the relationship can be broken. A(auxiliary system, electric servo system) = 0.5, A(auxiliary system, cooling system) = 0.5, A(auxiliary system, lubrication system) = 0.5, the electric servo system, auxiliary system, cooling system and lubrication system are clustered into auxiliary system. The retained modeling elements can be further optimized (MTC^(precision)^), MTC^(precision)^ = {CNC system, CNC controller, auxiliary system, PLC controller, cooling system, lubrication system, electric servo system, sensor, spindle drive, spindle motor}= {CNC system} + {auxiliary system}= {CNC controller, sensor, spindle motor, PLC controller} + {electric servo system, cooling system, lubrication system}. Here, the CNC system and auxiliary system are abstracted as subclasses of the third layer and clustered as control class.

**Figure 13:**
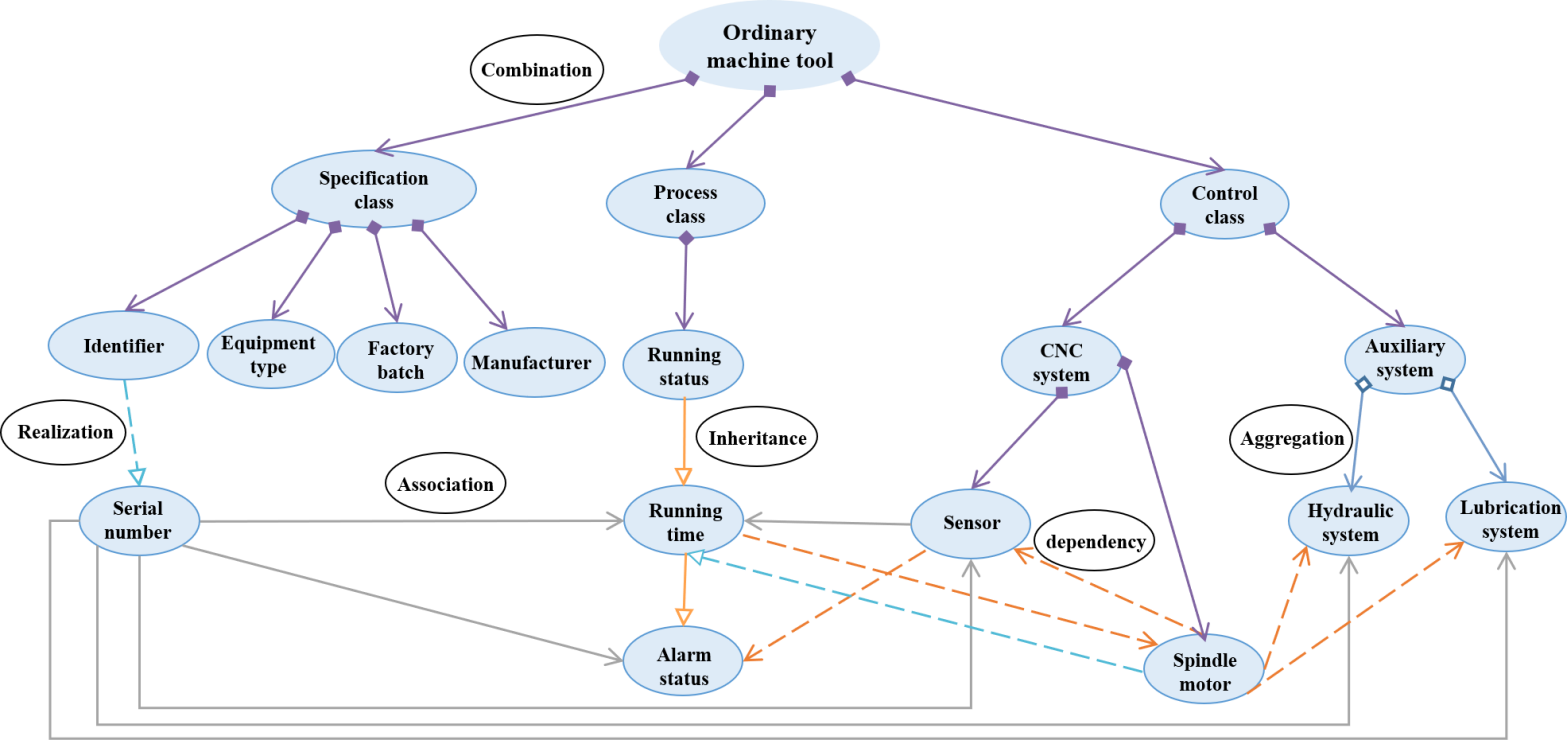
Information model of the ordinary machine tool.

**Figure 14:**
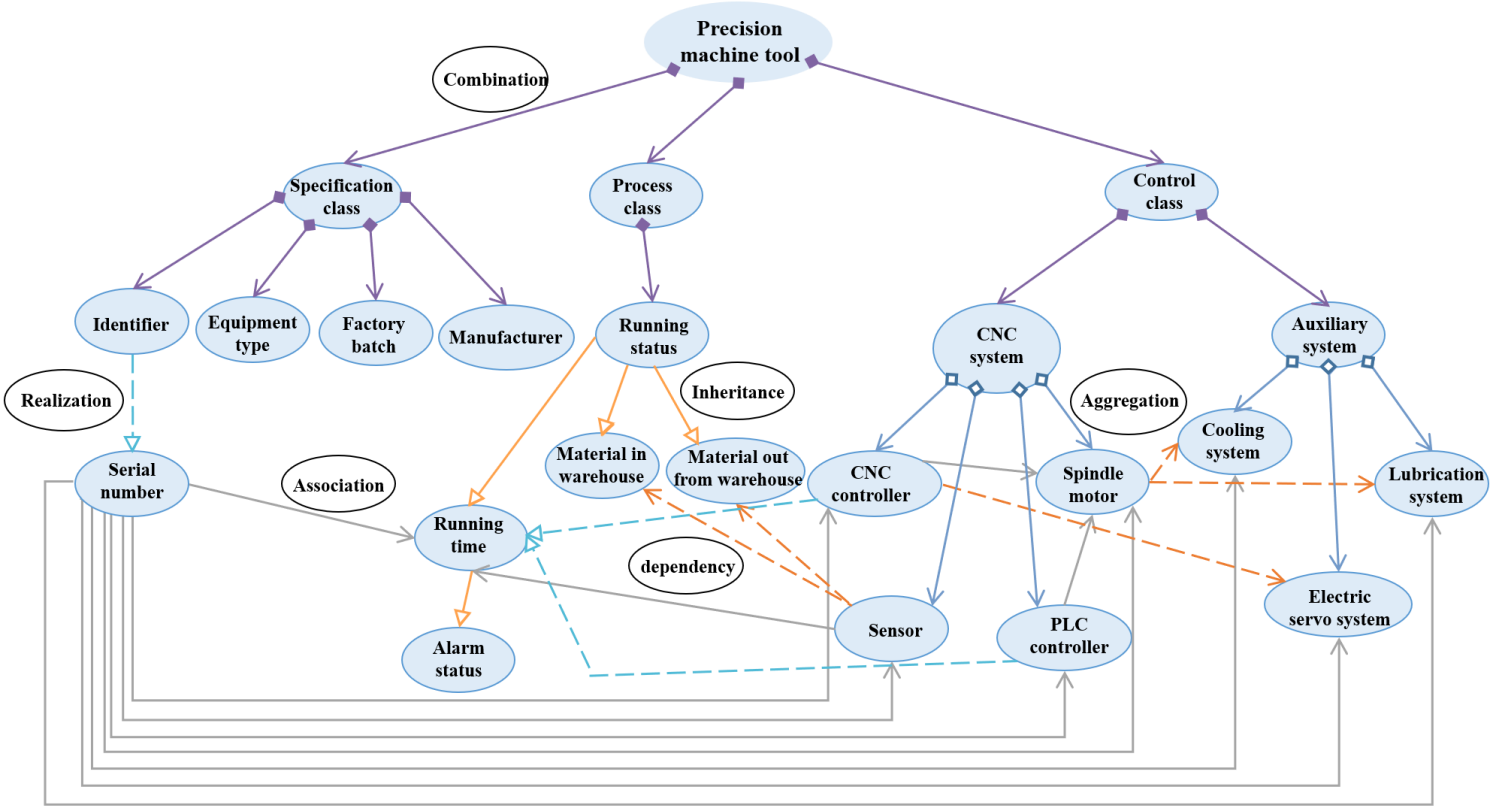
Information model of the precision machine tool.

After the information is extracted and organized, two 3IMs of ordinary and precision machine tools are represented as shown in Figs. 13 and 14. According to the optimized architecture, the modeling costs of ordinary and precision machine tools have saved 3 and 4 modeling elements, which accounts for 18.75% and 18.18% of the total, respectively.

### An application scene of a digital machining workshop

The research results of this paper have further been used in a digital machining workshop, which aims to realize data sharing and interoperability among different manufacturing systems. Because the semantic description and the data expression format from different manufacturers are not unified in the traditional workshop, various heterogeneities exist in the operating systems, communication interfaces, and hardware platforms. Considering the complexity of data communication, digital reformation of this kind of workshop needs a lot of workloads for project construction.

**Figure 15:**
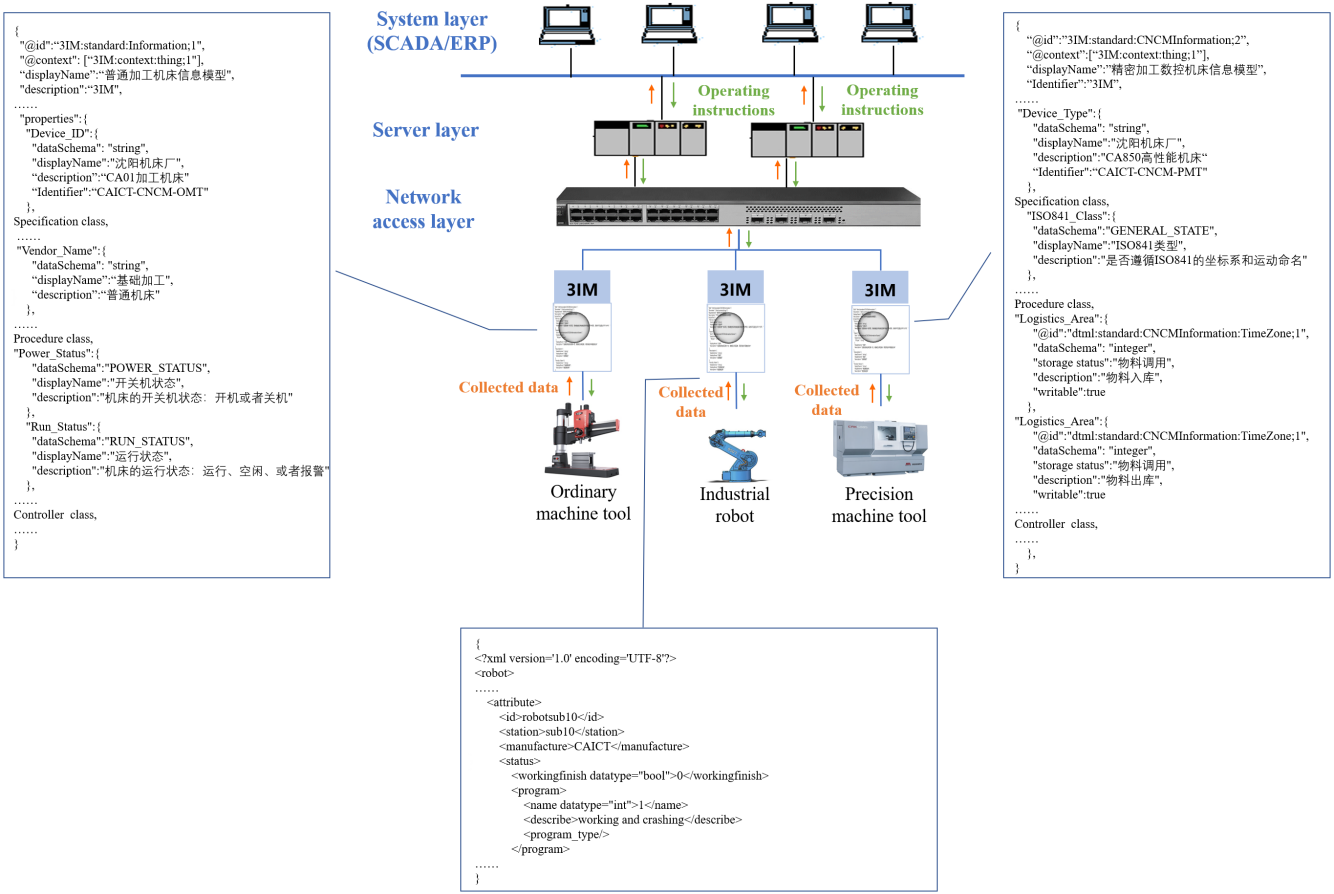
3IMs are used in a digital workshop.

With the proposed modeling method, several 3IMs are established for the machine tools, industrial robots and the system layer, as shown in Fig. 15. In this scenario, SCADA is responsible to collect variables from distributed devices, including device status, completion information, product quality data, etc. According to the configuration of the 3IMs, the data type, transmission parameters and return parameters of each variable are described in a united format, and then the collected data is forwarded to the server layer for local storage. Meanwhile, the authorization mechanisms are integrated with deployment of 3IM models, so that data in the heterogeneous devices can be mutually and trusted accessed and redrived in the closed working group. As tested, the on-site manufacturing data is collected and shared in real time, while the optimized operating instructions can be issued to devices without delay.

In the digital machining workshop, the 3IMs act as bridges of information interaction between on-site hardware equipment and management system, they are responsible for the data flow from different objects. Through a unified architecture and standard semantic description, the 3IMs provide a mutually recognized data format. In summary, the effectiveness of the modeling method is verified.

## Conclusions

This article firstly defined a special information model with seven key elements for the Industrial Internet as well as explained its concept and characteristics. Then a general modeling method was invented, which included information extraction, information organization, and model representation. By using a hybrid approach combining modeling value evaluation, semantic similarity and entropy, the information organization structure was optimized. As a new framework based on underground root structure was designed and six kinds of relations were introduced, the information model was represented and the maximum embodiment value of 3IM could be released. Finally, to verify the effectiveness of the modeling method, two specific 3IMs were built for the ordinary and precision machine tools. The study shows that the modeling costs of ordinary and precision machine tools have saved 3 and 4 modeling elements, which accounted for 18.75% and 18.18% of the total, respectively. As the 3IMs were applied in a digital machining workshop, the data exchange and interoperability are realized in the distributed systems even though they are heterogeneous.

The consideration of the information model plays a central role in the interoperability realization, technology development and standardization for Industrial Internet. This article only presented the modeling framework of 3IM from the aspect of methodology, however, due to the complex conditions in industrial scenes, some critical values, *e.g.*, thresholds for evaluation value and semantic similarity should be further devised, for example, using artificial intelligence (AI) algorithms while considering the statistics of the actual industrial requirements. Moreover, future work should emphasize on promoting the standardization and application of modeling technologies, and commit to the mutually agreed communication protocols and standardizing syntax and semantics as well.

##  Supplemental Information

10.7717/peerj-cs.1150/supp-1 Supplemental Information 1 The computer code of the ordinary machine tool

10.7717/peerj-cs.1150/supp-2 Supplemental Information 2 The computer code of the precision machine tool

10.7717/peerj-cs.1150/supp-3 Supplemental Information 3 The computer code of the industrial robot

10.7717/peerj-cs.1150/supp-4 Supplemental Information 4 Raw data used for calculating the uts1

10.7717/peerj-cs.1150/supp-5 Supplemental Information 5 Raw data used for calculating the uts2

10.7717/peerj-cs.1150/supp-6 Supplemental Information 6 Raw data used for modeling costs calculation

## Additional Information and Declarations

### Data Availability

The following information was supplied regarding data availability: The raw data and code are available in the Supplemental Files.
