# Supplementary material for: The proposal of a modeling methodology for an industrial internet information model
Source: PeerJ Comput Sci. 2022 Nov 15;8:e1150. doi: 10.7717/peerj-cs.1150 (PMC9680887; doi:10.7717/peerj-cs.1150)
Supplement: Supplemental Information 6 [file peerj-cs-08-1150-s006.docx]

The raw data following is used for modeling costs calculation.

| Num. | The original modeling elements of the ordinary machine tool | The original modeling elements of the precision machine tool | The optimized modeling element of the ordinary machine tool | The optimized modeling element of the precision machine tool |
| --- | --- | --- | --- | --- |
|  | manufacturer | manufacturer | manufacturer | manufacturer |
|  | factory batch | factory batch | factory batch | factory batch |
|  | serial number | serial number | serial number | serial number |
|  | equipment type | equipment type | equipment type | equipment type |
|  | production date | production date | running status | running status |
|  | running status | running status | running time | running time |
|  | running time | running time | alarm status | alarm status |
|  | alarm status | alarm status | CNC system | material in warehouse |
|  | working status | working status | auxiliary system | material out from warehouse |
|  | CNC system | material in warehouse | hydraulic system | CNC system |
|  | auxiliary system | material out from warehouse | lubrication system | CNC controller |
|  | hydraulic system | CNC system | spindle motor | sensor |
|  | lubrication system | CNC controller | sensor | spindle motor |
|  | spindle drive | sensor |  | PLC controller |
|  | spindle motor | spindle drive |  | auxiliary system |
|  | sensor | spindle motor |  | cooling system |
|  |  | PLC controller |  | lubrication system |
|  |  | auxiliary system |  | electric servo system |
|  |  | cooling system |  |  |
|  |  | lubrication system |  |  |
|  |  | electric servo system |  |  |
|  |  | hydraulic system |  |  |
| Total number | 16 | 22 | 13 | 18 |

For the ordinary machine tool, the saved modeling costs can be calculated as follows.

$$\frac{16-13}{16}\times100\%=18.75\%$$

For the ordinary machine tool, the saved modeling costs can be calculated as follows.

$$\frac{22-18}{22}\times100\%=18.18\%$$
